# Three new species of the genus *Sycophila* (Hymenoptera, Chalcidoidea, Eurytomidae) from China

**DOI:** 10.3897/zookeys.1029.60911

**Published:** 2021-04-08

**Authors:** Hui Xiao, Rui Zhang, Mengqing Gao

**Affiliations:** 1 Key Laboratory of Zoological Systematics and Evolution, Institute of Zoology, Chinese Academy of Sciences, Beijing, 100101, China Key Laboratory of Zoological Systematics and Evolution, Institute of Zoology, Chinese Academy of Sciences Beijing China; 2 Graduate School of Chinese Academy of Sciences, Beijing, 100049, China Graduate School of Chinese Academy of Sciences Beijing China; 3 Provincial Key Laboratory for Conservation and Utilization of Important Biological Resource in Anhui, College of Life Sciences, Anhui Normal University, Wuhu, 241000, China Anhui Normal University Wuhu China

**Keywords:** Chalcid wasp, fig wasp, key, parasitoids, plant galls, taxonomy

## Abstract

Three new species of *Sycophila* Walker (Hymenoptera, Eurytomidae), *S. hunanensis***sp. nov.**, *S. melanoloma***sp. nov.** and *S. melanopoda***sp. nov.**, are reported and described from mainland China. Meanwhile, *Plagiotrochus glaucus* Melika & Tang, 2011 (Hymenoptera, Cynipidae) is reported as a new host record of the genus *Sycophila*. A key to Chinese *Sycophila* and illustrations of external features of the species are provided.

## Introduction

*Sycophila* is one of the large genera in the family Eurytomidae. It was described for the first time by Walker in 1871 under the Agaonidae, with two species included (*S. megastigmoides* Walker, 1871 and *S. decatomoides* Walker, 1871), both reared from the fruits of *Ficus benghalensis* L.. Ashmead (1904) selected the latter as the type species of *Sycophila* and transferred the genus to the subfamily Idarninae in the Torymidae. Bouček (1974) transferred it to Eurytomidae and synonymized several genera with *Sycophila* (Bouček 1988). Since then, the genus has been extensively studied by several researchers, including Nieves Aldrey (1984), Narendran (1994) and Zerova (1995). The genus can be distinguished from other genera in the family Eurytomidae by the following combination of characters: marginal vein broadened, maculae dark brown and limited at the marginal and stigmal vein (or expanded to the disc), petiole elongate, and gaster often laterally compressed. Most species develop in very hard parts of plant galls or in figs, some species are recorded as parasitoids on gall makers (Bouček 1988; Narendran 1994; Chen et al. 1999; Lotfalizadeh and Gharali 2007). More than 100 different host species are reported for *Sycophila* (Noyes 2020), including species of Hymenoptera, Diptera, Lepidoptera and Hemiptera. Until now, 117 valid species of the genus have been described (Noyes 2020), but specific identification is difficult because the species differences are very small. Lotfalizadeh et al. (2008) used morphometrics and sequence data to distinguish several species based on morphological studies. It provides a new idea and method for the taxonomic study of this genus. Before the present work, only one species, *S. fujianensis* Özdikmen, was recorded from mainland China (Xu and He 2003; Özdikmen 2011). In this study, three new species, *S. hunanensis* sp. nov., *S. melanoloma* sp. nov. and *S. melanopoda* sp. nov. are reported and described from mainland China, meanwhile two species, *S. curta* Chen and *S. maculafacies* Chen, are newly recorded from mainland China.

## Materials and methods

All specimens were collected in the laboratory where they were reared from *Ficus microcarpa* L. and preserved in 75% or 95% ethanol. They were subsequently air-dried, point-mounted, and examined with a Leica MZ APO stereomicroscope. Photographs were taken under the Nikon Multizoom AZ100 system, and the plates were compiled using Adobe Photoshop software. Five species were identified, and all type specimens are deposited in the Institute of Zoology, Chinese Academy of Sciences, Beijing, China (**IZCAS**).

Morphological terminology follows that of Bouček (1988), Gibson et al. (1997) and Lotfalizadeh et al. (2007). All specimens were examined and identified based on the studies of Balduf (1932), Bouček (1988), Narendran (1994), Zerova (1995), Chen et al. (1999), Lotfalizadeh and Gharali (2007), Lotfalizadeh et al. (2008) and Zerova and Harten (2009). Body length (i.e., the length of body excluding the ovipositor sheaths) is measured in millimeters (mm), other measurements are given as ratios.

Abbreviations of morphological terms used are:

**Fu_n_** funicular 1, 2…

**POL** posterior ocellar distance

**OOL** ocellocular distance

**Gt_n_** gastral tergite 1, 2…

## Taxonomy

### Key to species

**Table:** 

1	Antenna slender, each funicular longer than broad, Fu_1_ at least 2.0× as long as broad	**2**
–	Antenna thick, Fu_1_ at most 1.5× as long as broad	**3**
2	Body yellow-brown, except collar of pronotum, median line of propodeum, and median area of Gt_1–3_ black; Fu_1_ 2.8× as long as broad, longer than pedicel; gastral petiole 4.0× as long as broad, Gt_4_ dorsally 1.3× as long as Gt_3_	***S. fujianensis***
–	Body yellow-brown except median line of gaster black; Fu_1_ 2.0× as long as broad, as long as pedicel; gastral petiole 3× as long as broad, Gt_4_ dorsally 1.8× as long as Gt_3_	***S. melanoloma* sp. nov.**
3	Body black, shoulder of pronotum and lateral panel of pronotum yellow-brown; fore wing with maculae not extending backward to disc; pedicel and flagellum combined longer than head width	***S. hunanensis* sp. nov.**
–	Body mainly yellow-brown; fore wing with maculae expending backward to disc; pedicel and flagellum combined as long as or shorter than head width	**4**
4	Pedicel and flagellum combined as long as head width; pronotum yellow-brown, thorax reddish brown, gaster dark brown	***S. melanopoda* sp. nov.**
–	Pedicel and flagellum combined shorter than head width; body yellow-brown	**5**
5	Fu_1_ 1.33× as long as broad, as long as pedicel; pronotum and mesosoma with sparsely umbilicate puncturation, scutellum without umbilicate puncturation	***S. curta***
–	Fu_1_ as long as broad, shorter than pedicel; pronotum, mesosoma and scutellum with sparsely umbilicate puncturation	***S. maculafacies***

#### Sycophila

Taxon classificationAnimaliaHymenopteraEurytomidae

Walker, 1871

CA570D0E-B3C5-5B1F-A07F-A62FF3A7843F

Sycophila Walker, 1871: 63. Type species: Sycophila decatomoides Walker, designated by Ashmead 1904; Bouček 1974: 267–268; Bouček 1988: 96–97; Narendran 1994: 156–170.
Tineomyza Rondani, 1872: 205. Type species: Tineomyza pistacina Rondani. Synonymized by Bouček 1974: 267–268.
Pseudisa Walker, 1875: 15. Type species: Pseudisa smicroides Walker. Synonymized by Bouček 1988: 96.
Isanisa Walker, 1875: 16. Type species: Isanisa decatomoides Walker. Synonymized by Bouček 1988: 96.
Decatomidea Ashmead, 1888: 42. Type species: Decatomidea xanthochroa Ashmead. Synonymized by Bouček 1988: 96.
Eudecatoma Ashmead, 1888: 42. Type species: Decatoma batotoides Ashmead, designated by Ashmead 1894. Synonymized by Bouček 1974: 267–268.

##### Diagnosis.

Body yellowish or brownish, occasionally black. Head wider than mesosoma, lower margin of clypeus bilobed. Antennal insertion slightly above or on lower ocular line, antennal formula 11153 in female, 11143 in male. Prothorax with pronotum rectangular, almost as long as mesoscutum; mesothorax dorsally convex, notauli deep and complete, scutellum convex; propodeum with an inverted V-shaped basal submedian carina. Fore wing with marginal vein broadened, mostly with dark brown maculae below marginal vein; postmarginal vein slightly shorter than marginal vein. Hind femur distinctly thickened. Petiole elongated, gaster compressed from side-to-side.

##### Biology.

Most species develop in plant galls or in figs, some extralimital species are recorded as parasitoids. The hosts involved Hymenoptera (Pteromalidae, Eulophidae, Eurytomidae, Tanaostigmatidae, Torymidae, Tenthredinidae, Cynipidae and Cecidomyiidae), Lepidoptera (Cecidosidae, and Gelechiidae), Diptera (Prodoxidae) and Hemiptera (Psyllidae) (Noyes 2020).

##### Distribution.

China (Hainan, Fujian, Hunan, Guangxi, Hongkong, Taiwan) (Luo et al. 1987; Huang et al. 1988; Beardsley 1998; Chen et al. 1999; Xu et al. 2002). The species of *Sycophila* are reported throughout the world (Narendran 1994; Noyes 2020).

#### Sycophila fujianensis

Taxon classificationAnimaliaHymenopteraEurytomidae

Özdikmen, 2011

177357EA-CC07-544B-8A4B-925702E2B15D

Sycophila fujianensis Özdikmen, 2011: 838. Replacement name for Sycophila flava Xu & He, 2003.
Sycophila flava Xu & He, 2003. Junior secondary homonym of Sycophila flava (Ashmead, 1881).

##### Diagnosis.

Body length 3.0 mm. Body yellow-brown in general except collar of pronotum, median line of propodeum, and median area of Gt_1–3_ black. Wings hyaline, marginal vein and the surrounding dark brown. Head in dorsal view 2.3× as wide as long, head in frontal view 1.2× as wide as high. Antennal insertion on lower ocular line, scrobes not reaching anterior ocellus. Antenna slender, each funicular longer than broad respectively; Fu_1_ 2.8× as long as broad, longer than the other funiculars; the length of the other funiculars shorter towards the end, the last funicular length 1.9× width. Fore wing 2.7× as long as broad, speculum distinct and closed behind; marginal vein 1.6× as long as stigmal vein; postmarginal vein shorter than marginal vein, 1.1× as long as stigmal vein. A row of long setae on the dorsal edge of hind tibia shorter than tibia width. Gaster cylindrical, 4.0× as long as broad, Gt_4_ dorsally 1.3× as long as Gt_3_; Length of Gt_4_ 1.2× length of Gt_3_. Male similar to the female, body length 3.0 mm, antenna with 4 funiculars; gaster short; petiole black dorsally.

##### Hosts.

Gall wasps (cynipids) in bamboo shoots.

##### Distribution.

China (Fujian) (Xu and He 2003).

#### Sycophila melanoloma

Taxon classificationAnimaliaHymenopteraEurytomidae

Zhang & Xiao sp. nov.

0473362E-BC9F-5ECD-82A0-C63D10000A1C

http://zoobank.org/B2CEEAEA-EB0A-4652-B314-74AE0A69B1C8

Figs 1–5

##### Material examined.

***Holotype*.** ♀, China: Hainan: Danzhou, 19.31°N, 109.34°E, VI.2006, reared form *Ficus microcarpa* L., leg. Haoyuan Hu. ***Paratypes*.** 4♀, same data as holotype.

##### Diagnosis.

Body slim, length 1.8–2.3 mm, mainly yellowish except eyes dark brown, median line of gaster black; antenna slender with Fu_1_ 2.0× as long as broad; fore wing hyaline, marginal vein enlarged and with dark brown maculae; gastral petiole longer than wide, gaster compressed laterally, dorsally arched.

##### Description.

**Figures 1–5.**
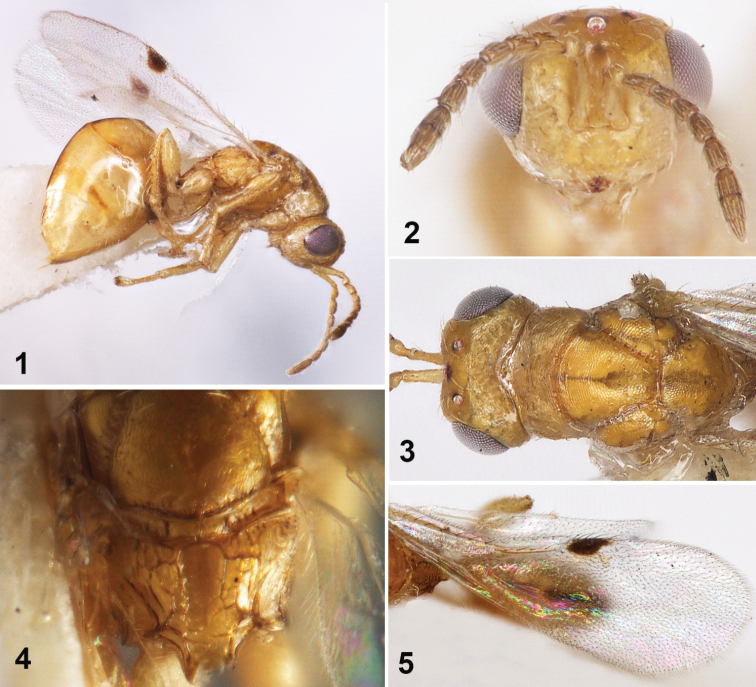
*Sycophila melanoloma* sp. nov., female holotype **1** body in lateral view **2** head in frontal view **3** head and mesosoma in dorsal view **4** propodeum in dorsal view **5** fore wing in dorsal view.

**Female (holotype). *Body*** (Fig. 1) length 2.0 mm. Body brownish yellow except eyes red-brown, median line of gaster black; antennae and legs concolorous with body; wings hyaline, venation yellow-brown except marginal vein enlarged with black maculae; head and thorax smooth, umbilicate puncturation sparse and shallow.

***Head*** with white pubescens sparse, head in frontal view 1.5× as wide as high (Fig. 2), eyes separated by 1.5× their height, malar space 0.7× eyes height, malar sulcus space 1.43× malar space. Antennal insertion above lower ocular line; scrobes deep and smooth, not reaching anterior ocellus, interantennal crest absent. Lower face smooth. Lower margin of clypeus with incision separating a single tooth on both sides; mandible three teethed. Head in dorsal view (Fig. 3) 1.67× as broad as long, occipital carina inconspicuous; temple length 0.2× eyes length; POL 2.5× OOL, OOL 2.0× ocellus diameter. Antennal formula 11153 (Figs 1, 2); scape reaching anterior ocellus, equal to eyes height, 5.0× as long as broad, 2.5× pedicel length; pedicel and flagellum combined 1.33× head width; pedicel in lateral view 2.0× as long as broad, equal to Fu_1_; anellus 0.5× as long as broad; Fu_1_ 2.0× as long as broad, Fu_2_-Fu_5_ slightly shorter than Fu_1_ (1.85× as long as broad); clava length 3.0× width, shorter than the following three funiculars combined; each funicular with single row of sensilla; ventral surface of clava without micropilose area.

***Mesosoma*** 1.45× as long as broad in dorsal view. Pronotum 0.46× as long as broad. Mesoscutum 0.62× as long as broad, notauli deep and complete. Scutellum as long as broad. Propodeum shorter than mesoscutum (0.39×), nucha distinct, median carina and plica absent; median longitudinal furrow distinct, irregular cells formed by irregular ridges on both sides (Fig. 4). Fore wing (Fig. 5) 2.86× as long as broad, with marginal fringe; maculae dark brown, confining on marginal vein and stigmal vein, not extending backward; marginal vein triangular broadened; ratio of marginal vein: postmarginal vein: stigmal vein as 8:3:3. Fore femur stout, fore tibia with a ventral spur at apex; mid femur enlarged, mid tibia thin and outer edge with a single row of setae; hind coxa stout, 2.0× as long as broad; hind femur enlarged, 1.67× as long as broad; hind tibia with 2 ventral spurs at apex.

***Metasoma*** 1.67× as long as mesosoma. Gaster petiolate, 3.0× as long as broad, laterally compressed. Gaster bare and smooth, distinctly compressed and dorsally arched, median line of gaster black; length of Gt_4_ as long as Gt_1_-Gt_3_ combined, 2.0× as Gt_3_. Ovipositor exposed, 0.08× as long as gaster.

**Male.** Unknown.

##### Etymology.

The specific name is derived from the Latin ’melanolomus’, referencing the character of the gaster with a median black line.

##### Remarks.

The species is similar to *S. petiolata* Chen, 1999 from Taiwan (Chen et al. 1999) but noticeably different by the gaster compressed and dorsally arched (gaster oval in *S. petiolata*), mesonotum and metanotum yellowish (mesonotum and metanotum with dark brown patches in *S. petiolata*).

##### Host.

*Ficus microcarpa* L.

##### Distribution.

China (Hainan).

#### Sycophila hunanensis

Taxon classificationAnimaliaHymenopteraEurytomidae

Xiao & Gao sp. nov.

CCCC8108-8F5D-5240-A74F-CF399B86E243

http://zoobank.org/E97CFFFB-03C1-42AD-84FE-FFE09135E9C0

Figs 6–13

##### Material examined.

***Holotype*.** ♀, China: Hunan: Yanling Xian: Shidu, 1.III.2017, ex. galls of *Plagiotrochus glaucus* Melika & Tang (Cynipini), leg. Gaozhi Zhao. ***Paratype*.** 4♀1♂, same data as holotype; 2♂, China: Hainan: Wuzhi Shan, 708-1206M, 9.IV.2010, leg. Tianyang Jiao.

##### Diagnosis.

Body length 1.8–2.0 mm, mainly black except lateral shoulder and lateral panel of pronotum yellow-brown; antenna slightly thick, Fu_1_ 1.23× as long as broad, Fu_2_-Fu_5_ subequal to Fu_1_; pedicel and flagellum combined slightly longer than head width (1.1×); marginal vein enlarged, maculae not extending backward to disc of fore wing.

##### Description.

**Figures 6–13.**
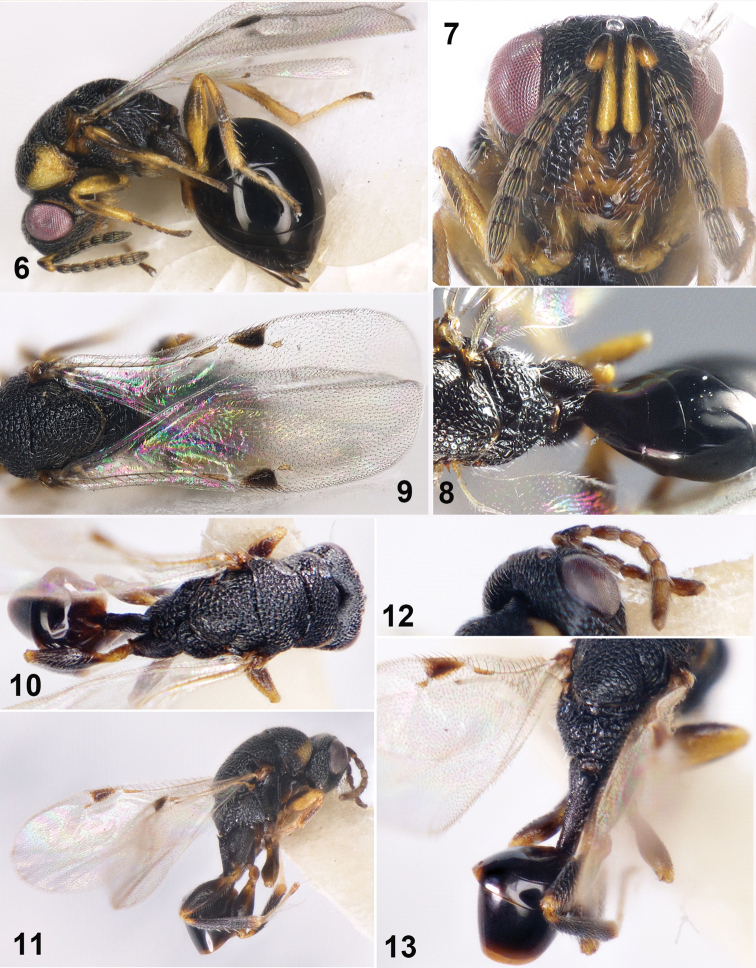
*Sycophila hunanensis* sp. nov. **6–9** female holotype **6** body in lateral view **7** head in frontal view **8** propodeum in dorsal view **9** fore wing in dorsal view **10–13** male **10** body in dorsal view **11** body in lateral view **12** antenna **13** propodeum, petiole and gaster in dorsal view.

**Female (holotype)**. ***Body*** (Fig. 6) length 2.0 mm, body black except eyes red-brown, middle part of lower face yellowish, shoulder of pronotum and lateral panel of pronotum yellow-brown. Antennae brown except scape yellowish and pedicel yellow-brown. Legs yellowish except coax dark brown, femur and tibia brown on middle part. Wings hyaline, venation yellow-brown except marginal vein enlarged with black spot. Head and thorax with densely umbilicate puncturation.

***Head*** hairy, 1.25× as wide as high in frontal view (Fig. 7), eyes separated by 1.52× their height, malar space 0.81× eyes height. Antennal insertion on lower ocular line; scrobes deep and smooth, not reaching anterior ocellus, interantennal crest absent. Umbilicate puncturation shallow on lower face. Lower margin of clypeus with incision separating a single tooth on both sides; mandible three teethed. Head in dorsal view 1.67× as wide as long, occipital carina inconspicuous; temple length 0.3× eyes length; POL 2.16× OOL, OOL 2.0× ocellus diameter. Antennal formula 11153; scape reaching anterior ocellus, equal to or slightly shorter than eyes height, 6.25× as long as broad, 2.36× pedicel length; pedicel and flagellum combined 1.1× head width; pedicel in lateral view 2.0× as long as broad, longer than Fu_1_; anellus 0.5× as long as broad; Fu_1_ 1.23× as long as broad, Fu_2_-Fu_5_ as long as Fu_1_; clava length 3.0× width, shorter than the following three funiculars combined; each funicular with a single row of sensilla; ventral surface of clava without micropilose area.

***Mesosoma*** 1.58× as long as broad. Pronotum 0.51× as long as broad. Mesoscutum 0.67× as long as broad, notauli shallow and complete. Scutellum slightly longer than broad (1.11×). Propodeum rugosity (Fig. 8), shorter than mesoscutum (0.73×); basal sculpture of median furrow with one row of areoles; median carina and plica absent. Fore wing (Fig. 9) 2.3× as long as broad, with marginal fringe, speculum and basal hairline; maculae dark brown, confining on marginal vein and stigmal vein, not extending backward; marginal vein triangular broadened; ratio of marginal vein: postmarginal vein: stigmal vein as 12:2:10. Hind coxa stout, 2.0× as long as broad; hind femur enlarged in middle part, 3.33× as long as broad; hind tibia with 2 ventral spurs.

***Metasoma*** 1.22× as long as mesosoma. Gaster with petiole longer than broad, reticulate; gaster 2.0× as long as broad, laterally compressed. Gaster arched in lateral view, gastral tergum smooth; Gt_4_ longest, 1.36× as long as Gt_3_. Ovipositor not exposed.

**Male.** Length 2.0 mm, body (Figs 10, 11) black except tegula, anterior corner of pronotum yellowish, apex of femur, apex of tibia and tarsus yellowish. Head and thorax with densely umbilicate puncturation. Antenna dark brown, formula 11143 (Fig. 12), Fu_1_ 1.83× as long as broad, Fu_2_-Fu_4_ equal to Fu_1_. Petiole (Fig. 13) 4.0× as long as broad, shorter than gaster; gaster 1.55× as long as petioles, Gt_4_ longer than other tergites.

##### Etymology.

Named after the location of the type material.

##### Remarks.

The species is similar to *S. biguttata* (Swederus, 1795) from Sweden (Lotfalizadeh and Gharali 2007), but different by the maculae on fore wing not extending backward to disc, hind tibia pale yellowish except mid part brown.

##### Host.

The wasps were reared from the galls of *Plagiotrochus glaucus* Tang & Melika, 2011 (Hym., Cynipidae) (Tang and Melika 2011) in China.

##### Distribution.

China (Hunan, Hainan).

#### Sycophila melanopoda

Taxon classificationAnimaliaHymenopteraEurytomidae

Zhang & Xiao sp. nov.

F4D865ED-23B1-522C-BFF7-ACEA6136A7B2

http://zoobank.org/7BDF6F62-524C-4103-9CBC-58B1527A5162

Figs 14–18

##### Material examined.

***Holotype*.** ♀, China: Hainan: Danzhou, 19.31°N, 109.34°E, VI.2006, reared form *Ficus microcarpa* L., leg. Haoyuan Hu. ***Paratypes*.** ♀, same data as holotype.

##### Diagnosis.

Body length 1.6–1.8 mm, head and mesonotum brownish black, pronotum yellow, gaster dark brown; antenna with pedicel and flagellum combined as long as head width, Fu_1_ 1.6× as long as broad. Fore wing with maculae expending backward to disc, marginal vein 2.5× as long as postmarginal vein, postmarginal vein shorter than stigmal vein. Hind femur enlarged, 1.5× as long as broad, gaster compressed.

##### Description.

**Figures 14–18.**
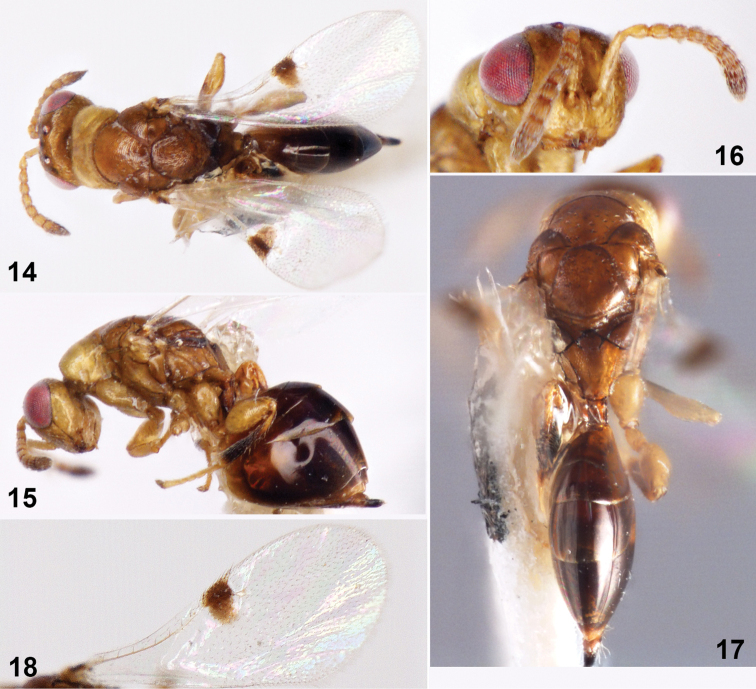
*Sycophila melanopoda* sp. nov., female holotype **14** body in dorsal view **15** body in lateral view **16** head in frontal view **17** mesosoma and metasoma in dorsal view **18** fore wing in dorsal view.

**Female (holotype). *Body*** length 1.8 mm (Figs 14, 15). Head and mesonotum brownish black, pronotum yellow, gaster dark brown; forehead black, gena yellow, eyes red-black; antennal yellow except clava dark brown; legs yellow except hind tibia concolorous with gaster; wings hyaline, venation yellow-brown; marginal vein enlarged, maculae expanded backward. Head and thorax with sparsely shallower umbilicate puncturation.

***Head*** in frontal view 1.17× as wide as high. Face with white pubescens sparse; eyes separated by 0.94× their height; malar space 0.35× eyes height; malar sulcus space 1.33× malar space. Antennal insertion slightly above lower ocular line, at 3/4 of head height. Lower margin of clypeus emarginated and with a small tooth on both sides; mandible three teethed. Head in dorsal view 2.15× as wide as high, occipital carina inconspicuous; temple length 0.33× eyes length; POL 2.25× OOL, OOL 4.0× ocellus diameter. Antennal formula 11153 (Fig. 16); scape not reaching anterior ocellus, 5.5× as long as broad, 0.65× eyes height, 3.67× pedicel length; length of pedicel and flagellum combined as long as head width; pedicel in lateral view 2.0× as long as broad, as long as Fu_1_; anellus 0.5× as long as broad; Fu_1_ 1.6× as long as broad, following funiculars sincrease gradually on length and width; clava length 1.75× width, shorter than the following three funiculars combined; each funicular with a single row of sensilla; ventral surface of clava without micropilose area.

***Mesosoma*** 1.62× as long as broad, with reticulation and sparsely umbilicate puncturation in dorsal view. Pronotum near rectangle, 0.42× as long as broad. Mesoscutum 0.58× as long as broad; notauli deep and complete. Scutellum as long as broad, medially protuberate. Propodeum (Fig. 17) 0.47× as long as mesoscutum, with small and dense sculpture; with an inverted V-shaped carina and a V-shaped carina, plica distinct, median carina absent. Fore wing (Fig. 18) 2.34× as long as broad, maculae expending backward to disc, marginal vein subparallel; ratio of marginal vein: postmarginal vein: stigmal vein as 5:2:3. Fore tibia with a ventral split spur at apex; mid tibia slim, with a ventral spur at apex. Hind coxa enlarged, 2.5× as long as broad; hind femur enlarged, 1.5× as long as broad; hind tibia ventrally with single apical spur.

***Metasoma*** 1.19× as long as mesosoma. Gaster petiolate; gaster near rhombus, 2.33× as long as broad; gastral tergum smooth; Gt_4_ longest, 1.25× as long as Gt_3_. Ovipositor exposed, 0.17× as long as gaster.

**Male.** Unknown.

##### Etymology.

The specific name is derived from the Latin ‘*melano*’ (black) and ’*podus*’ (foot), referencing the character of hind tibia black.

##### Remarks.

The species is similar to *S. maculafacies* Chen, 1999 from Taiwan (Chen et al. 1999) but noticeably different by pedicel and flagellum combined as long as head width (shorter than head width in *S. maculafacies*), hind tibia black (yellow-brown in *S. maculafacies*), gaster dark brown (yellow-brown in *S. maculafacies*).

##### Host.

*Ficus microcarpa* L.

##### Distribution.

China (Hainan).

#### Sycophila curta

Taxon classificationAnimaliaHymenopteraEurytomidae

Chen, 1999

CED505FF-E184-56D7-8F61-B79FA3A79C90

Figs 19–22

Sycophila curta Chen, 1999, in Chen et al. 1999: 45.

##### Material examined.

3♀, China: Hainan: Danzhou, VI.2006, leg. Haoyuan Hu. 5♀, China: Hainan: Danzhou, VIII.2006, leg. Haoyuan Hu. 4♀, China: Hainan: Lingshui, IV.2005, leg. Yanzhou Zhang, Tongxin Zhang. ♀, China: Guangxi: Wuzhou, X.2005, leg. Yanzhou Zhang, Wei Li.

##### Diagnosis.

**Figures 19–22.**
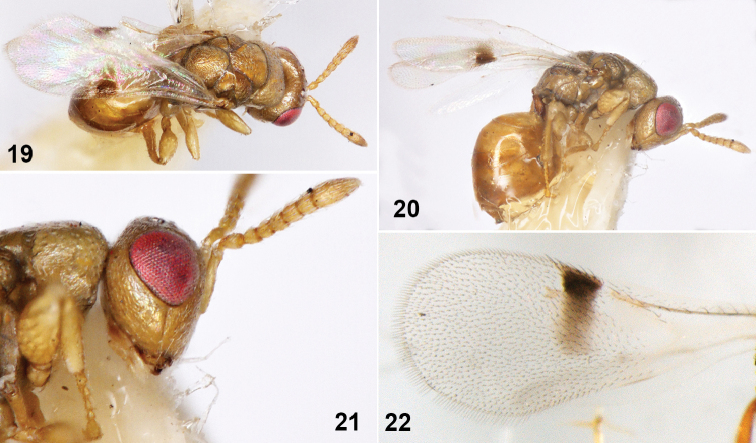
*Sycophila curta* Chen, female **19** body in dorsal view **20** body in lateral view **21** head in lateral view **22** fore wing in dorsal view.

Body (Figs 19, 20) length 1.38–1.8 mm. Body yellow-brown, eyes reddish, antennal yellow, wings venation yellow-brown except marginal vein enlarged and with dark brown maculae. Head in frontal view 1.4× as wide as high, eyes separated by 1.5× their height; malar space 0.6× eyes height. Lower margin of clypeus emarginated and with a small tooth on both sides, mandible three teethed. Head in dorsal view 2.14× as broad as long, POL 3.33× OOL, OOL 3× ocellus diameter. Antennal insertion on lower ocular line; scape 4.5× as long as broad, not reaching anterior ocellus (Fig. 21); scape length shorter than eyes height; pedicel as long as Fu_1_; length of pedicel and flagellum combined shorter than head width (0.83×); Fu_1_ 1.33× as long as broad. Pronotum and mesosoma with sparsely umbilicate puncturation, scutellum sub-rectangular, without umbilicate puncturation. Fore wing (Fig. 22) with maculae around marginal, postmarginal and stigmal vein, and expending backward to disc. Marginal vein broaden, stigma elongate; marginal vein 1.5× as long as postmarginal vein, 3.0× as long as stigmal vein. Legs covered with soft small setae. Gaster blob-shape. Ovipositor unexposed.

**Male.** No male species was found in this study. According to Chen et al. (1999), body length 1.14–1.62 mm. Body yellowish, eyes dark red, 2 brown black spots at the end of gaster. Body covered with small umbilicate puncturation. Head as wide as high. Antennal formula 11143. Petiole shorter than gaster.

##### Host.

*Ficus microcarpa* L.

##### Distribution.

China (Guangxi, Hainan, Taiwan).

#### Sycophila maculafacies

Taxon classificationAnimaliaHymenopteraEurytomidae

Chen, 1999

F2971949-2090-5437-8FF2-C107C8A9A0EA

Figs 23–26

Sycophila maculafacies Chen, 1999, in Chen et al. 1999: 51.

##### Material examined.

5♀, China: Hainan: Zhanzhou, V. 2006, leg. Haoyuan Hu. 6♀, China: Hainan: Zhanzhou, VI. 2006, leg. Haoyuan Hu. 5♀, China: Hainan: Zhanzhou, VIII.2006, leg. Haoyuan Hu. 5♀, China: Hainan: Zhanzhou, IX.2006, leg. Haoyuan Hu. 4♀, China: Hainan: Lingshui, IV.2005, leg. Yanzhou Zhang, Tongxin Zhang. ♀, China: Guangxi: Wuzhou, X.2005, leg. Yanzhou Zhang, Wei Li.

##### Diagnosis.

**Figures 23–26.**
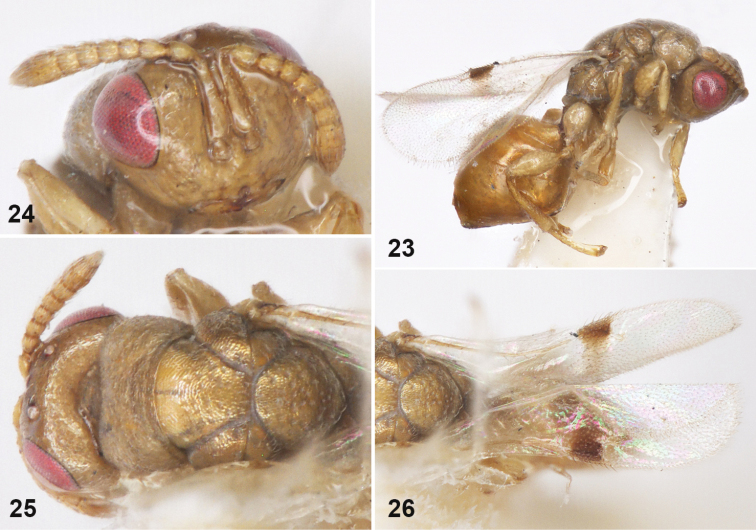
*Sycophila maculafacies* Chen, female **23** body in lateral view **24** head and antenna in frontal view **25** head and thorax in dorsal view **26** fore wing in dorsal view.

**Female.** Body (Fig. 23) length 1.5–1.62 mm. Body yellow-brown (or head, thorax, and gaster dark brown except pronotum yellowish); eyes dark red; antennal yellow or yellow-brown; wings hyaline, venation yellow-brown except marginal vein enlarged and with dark brown maculae; head and thorax with sparsely shallower umbilicate puncturation. Head in frontal view 1.25× as wide as high (Fig. 24), eyes separated by 1.6× their height; malar space 0.8× eyes height. Head in dorsal view 2.0× as broad as long, POL 5× OOL, OOL 3.0× ocellus diameter. Antennal insertion on lower ocular line. Antenna stout, formula 11153 (11143 in male) (Figs 24, 25), scape 5.0× as long as broad, not reaching anterior ocellus; scape length equal to eyes height, 3.33× as long as pedicel; pedicel slightly longer than Fu_1_; each funicular square or shorter than its broad, (Fu_1_ square, Fu_2_ 0.78× as long as broad); pedicel and flagellum combined shorter than head width (0.88×). Mandible three teethed. Mesosoma (Fig. 25) 1.4× as long as broad, pronotum and mesosoma with sparsely umbilicate puncturation, notauli deep and complete, scutellum as long as broad. Fore wing (Fig. 26) marginal vein broaden, stigma elongate; marginal vein as long as stigmal vein, 1.43× as long as postmarginal vein. Gaster diamond-shape in dorsal view.

**Male.** According to Chen et al. (1999), body length 0.96–1.20 mm. Frons black, gena yellow, eyes dark red, body black except pronotum yellowish. Body covered with small umbilicate puncturation. Head as wide as high, antennal formula 11143. Petiole shorter than gaster.

##### Host.

*Ficus microcarpa* L.

##### Distribution.

China (Guangxi, Hainan, Taiwan).

## Supplementary Material

XML Treatment for Sycophila

XML Treatment for Sycophila fujianensis

XML Treatment for Sycophila melanoloma

XML Treatment for Sycophila hunanensis

XML Treatment for Sycophila melanopoda

XML Treatment for Sycophila curta

XML Treatment for Sycophila maculafacies
